# Arthralgias, fatigue, paresthesias and visceral pain: can joint hypermobility solve the puzzle? A case report

**DOI:** 10.1186/s12891-016-0905-2

**Published:** 2016-02-04

**Authors:** Marco Folci, Franco Capsoni

**Affiliations:** Allergy, Clinical Immunology and Rheumatology Unit, IRCCS Istituto Auxologico Italiano, Piazzale Brescia 20, 20149 Milan, Italy; University of Milan, Milan, Italy

**Keywords:** Joint hypermobility syndrome, Arthralgia, Fatigue, Paresthesias, Beighton score

## Abstract

**Background:**

Joint hypermobility syndrome describes a disorder in which musculoskeletal pain occurs in a generalized joint hypermobility substrate. The clinical picture comprises variable manifestations which involve mainly but not exclusively the musculoskeletal system, and evolve over the person’s lifetime.

**Case presentation:**

Describing the case of a 20-year-old female with generalized arthro*-*myalgias, persistent fatigue and troublesome visceral pain, we illustrate how a frequently ignored clinical sign such as joint hypermobility can be the keystone to clarify different simultaneous symptoms. All of the patient’s physical complaints had been investigated separately during her previous medical examinations, and several tests repeatedly gave negative results. The patient received different diagnoses that describe only part of her problems, such as irritable bowel syndrome for visceral pain, fibromyalgia for arthralgias or depression for fatigue. These approaches gave rise to pharmacological or physical treatments which did not improve her quality of life in any way and in some instances worsened the situation. Pronounced joint hypermobility which led the patient to flex her joints excessively, causing subluxations in several districts, was the only sign overlooked.

**Conclusion:**

Exploring the patient’s articular features in her clinical context led us to diagnose joint hypermobility syndrome, a complex and often ignored condition. The case highlights the utility of a multidisciplinary approach and coordinated interventions to define and manage this clinical entity.

## Background

Joint hypermobility (JH) is defined as the ability to move specific articular segments painlessly beyond the normal range. A localized JH may be seen in some professions [1], or a generalized one which usually results from a congenital disease or acquired conditions. Whereas the latter is common among athletes [2], congenital JH may be an indicator of potentially threatening connective tissue diseases, such as Marfan syndrome (MFS) or Ehlers-Danlos syndrome (EDS) in particular, though most patients appear with no signs of further serious conditions.

JH is a relatively common finding (from 10 to 30 % of children) [3] and present in addition to a wide range of subtle symptoms such as musculoskeletal and neuropathic pain, joint instability, fatigue or anxiety [4, 5], but also cardiovascular autonomic diseases [6, 7] and bowel disturbance [8, 9] which are usually investigated and treated as distinct problems. JH and some of these features define the picture of the so-called joint hypermobility syndrome (JHS), a wide-spectrum clinical condition that is often hard to diagnose. It is a challenge to assess the frequency of JHS in the general population; the undefined signs, the lack of specific laboratory findings, but also the patients’ difficulty in describing their symptoms make it easy to underestimate or overlook this clinical picture. Moreover the syndrome is very similar – sometimes identical - to the more common variant of EDS hypermobility type (EDS-HT) (rev in [10–12]).

**Fig. 1:**
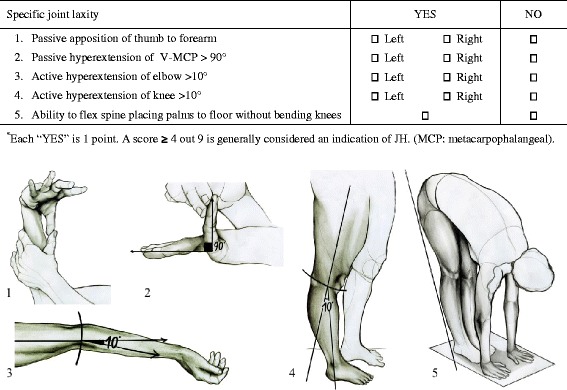
Beighton score for joint laxity^*^

Even though JHS presents the clinician with a substantial challenge, a pivotal issue in reaching a diagnosis is proper investigation. Hypermobility can be measured with a standardized method but the main problem is how to relate hypermobile patients to the clinical entity. The Beighton hypermobility score [13] is the most widely used system for rating hypermobility of the peripheral joints and spine (Fig. 1) and even if it does not consider pauci-articular conditions or other sites, such as the foot, it can detect the majority of cases. The Brighton criteria [14], which incorporate the Beighton score as well as the main symptoms and phenotypic features, are used to diagnose JHS (Table 1). A very effective five-point questionnaire was recently proposed by Hakim and Grahame to evaluate JH [15] (Table 2).

**Table 1:** Revised Brighton diagnostic criteria

*Major Criteria*
1. Beighton score of 4/9 or greater
2. Arthralgia for more than 3 months in 4 or more joints
*Minor Criteria*
1. A Beighton score of 1, 2 or 3/9 (0 to 3 if over age 50)
2. Arthralgia for more than 3 months in 1–3 joints, or back pain ≥3 months, or spondylosis, spondylolysis, spondylolisthesis
3. Dislocation or subluxation in more than one joint, or in one joint on more than one occasion
4. Soft tissue rheumatism in 3 or more locations (eg, epicondylitis, tenosynovitis, bursitis)
5. Marfanoid habitus
6. Abnormal skin (eg, striae, hyperextensible, thin, or papyraceous scarring)
7. Eye abnormalities (eg, drooping eyelids, myopia, anti mongoloid slant)
8. Varicose veins or hernia or uterine/rectal prolapse
JHS is diagnosed in three cases, if present: I) Two major criteria; II) One major and two minor criteria; III) Four minor criteria. The disorder is excluded in those patients with MFS or EDS.

**Table 2:** Five point Hypermobility questionnaire

1. Can you now [or could you ever] place your hands flat on the floor without bending knees?
2. Can you now [or could you ever] bend your thumb to touch your forearm?
3. As a child, did you amuse your friends by contorting your body into strange shapes or could you do the splits?
4. As a child or teenager, did your kneecap or shoulder dislocate on more than one occasion?
5. Do you consider yourself “double-jointed”?
Answering yes to 2 or more of these questions suggests hypermobility (sens 85 %, spec 90 %)

## Case presentation

A 20-year-old Caucasian female was referred to our out-patient rheumatology unit for generalized arthralgia and myalgia, associated with persistent fatigue. She reported recurrent sprains and tendonitis in the wrists and ankles during sport since childhood but nevertheless she practiced skiing at a competition level. In 2008, when she was 14, she had developed Raynaud’s phenomenon which was promptly evaluated by a rheumatologist and classified as a primary condition on the basis of a completely normal capillaroscopy and complete blood tests. The year after this, while the patient was in the United States, she caught first of all SARS flu, and then Epstein-Barr virus which resulted in a long period of persistent asthenia that kept her bedridden for some months.

In 2010, she started physical training again, but reported repeated musculoskeletal traumatic events, responding poorly to physical therapy. The worst episode was a neck trauma due to a severe skiing fall which caused an inversion of the natural cervical spine lordosis. This worsened the patient’s fatigue and aggravated the diffuse arthralgia. Gradually the previous symptoms were joined by lower limb paresthesia and in 2011 she was investigated for a suspected neurological or muscle disease (brain MRI, four-limbs EMG), but the result was negative. Also negative was MRI of the entire spine, a total body bone scan, and a skull and jaws X-ray carried out at short intervals. She was seen twice by rheumatologists, and both concluded with a diagnosis of fibromyalgic syndrome even if there was no subsequent confirmation at rheumatology control visits.

Between 2011 and 2013, the patient tried to pick up her physical activity once more, but encountered difficulties in running and swimming, and eventually stopped because of the worsening of joint pain. At the same time she complained of sleep disturbance, poor concentration and mood depression. She was therefore examined by a neurologist and after a few months she was treated with clonazepam which did not relieve her symptoms but, despite that she was still following the therapy when she attended our unit. The clinical picture, diagnostic procedures, therapies prescribed and their results are summarized in Table 3.

**Table 3:**
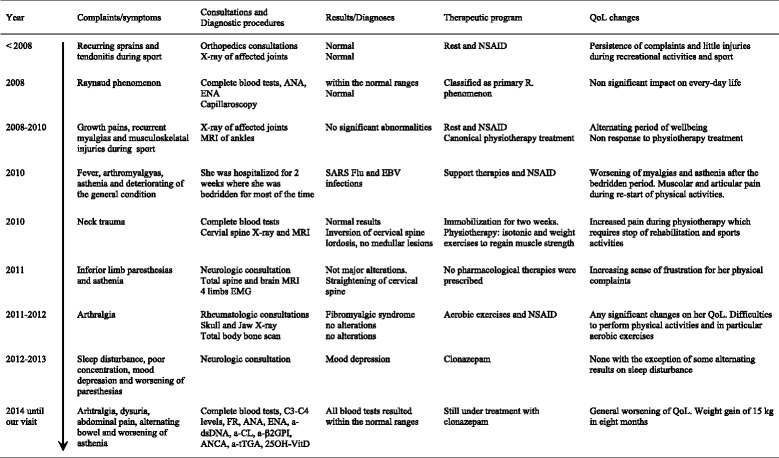
Timeline of patient clinical history, diagnostic procedures and therapeutic programs

Abbreviations: *Abs* autoantibodies, *ANA* anti-nuclear Abs, *ENA* anti-extractable nuclear antigen Abs, *a-dsDNA* anti-double strand DNA Abs, *a-CL* anti-cardiolipin Abs, *a-B2GPI* anti-B2 glicoprotein I Abs, *ANCA* anti-neutrophil cytoplasmic Abs, *a-tTGA* anti-tissue transglutaminase Abs, *RF* rheumatoid factor, *MRI* magnetic resonance imaging, *EMG* electromyography, *NSAID* non steroideal antinflammatory drugs, *Complete blood tests* refers to: blood cell count, liver and kidney function, serum electrolytes, serum protein electrophoresis, acute phase reactants, serum IgA, IgM and IgG levels, thyroid function, urine analysis; *QoL* quality of life

At our examination in July 2014, the patient appeared in good general condition. She complained of diffuse arthralgia mainly in the shoulders, ankles, wrists and knees, which had a heavy impact on her life. She said that these symptoms became worse during physical effort, so she was obliged to stop what she was doing until the symptoms passed, causing a complete change in her lifestyle. She reported a weight gain of about 15 kg in the last 8 months, which she explained by the interruption of physical activities. She reported variable abdominal discomfort, alternating bowel and sometimes difficult urination, with dysuria and pollakiuria although she had no urinary infections. In addition, she described chewing problems which gave rise to moderate pain in the temporo-mandibular joints. The series of symptoms and functional limitations created a feeling of frustration, though not true depression.

Her latest laboratory investigation indicated normal blood cell count, kidney, liver and thyroid function, acute phase reactants and C3-C4 levels. A complete autoantibodies (Abs) panel (anti-nuclear Abs, anti-extractable nuclear antigen Abs, anti-double strand DNA Abs, anti-cardiolipin Abs and anti-neutrophil cytoplasmic Abs) was all negative. At presentation pulmonary, cardiovascular and abdominal examinations gave normal findings. Peripheral pulses were normal. The pupils reacted normally to light and accommodation. Superficial lymph nodes were not detectable. Muscle tone and strength were apparently within normal limits and osteo-tendinous reflexes were present. The skin appeared thin and elastic, without scarring.

Joint examination detected no swelling or other signs of inflammation. Axial and peripheral joint mobility was preserved, with widespread joint hypermobility: she had evident extra-range mobility of the knees, elbows, fingers (strongly hyper-extended) at both active and passive mobilization. The patient could stretch and twist her thoracic-lumbar spine but could also dislocate the temporo-mandibular joint with extreme facility; she reported she had been able to do the splits as a child. We measured the range of motion with a goniometer to establish the precise articular extensibility. Then we applied a Beighton score with a result of 6/9, indicative of joint hypermobility.

On the basis of clinical history, previous investigations and physical examination we excluded clinical entities such as celiac disease, fibromyalgia or other connective tissue disorders and suggested a diagnosis of JHS as two of the main Brighton diagnostic criteria were met (Beighton score 4/9 or more; arthralgia for longer than 3 months in four or more joints) and the Hakim and Grahame questionnaire (positive answers to questions 1, 2 and 3).

## Conclusion

The term JHS was first used by Kirk and his group in 1967 to describe a disorder in which musculoskeletal pain occurs in a generalized joint hypermobility substrate [16]. Most authorities now are interpreting JHS as a hereditable connective tissue disorder - as proved by studies on twins [17] and the role of the tenascin X gene [18]. Tenascin X, an extracellular matrix protein, seems to be low in about 40 % of people who clinically present JH, arthralgia and abnormal skin [18]; moreover, 5–10 % of patients diagnosed with JHS or EDS-HT have low serum levels of tenascin X [18]. Despite this, virtually no genes have been found to be strictly related to JHS - with some exceptions of familial clusters [19].

The main feature of JHS and probably the most striking is the ability to flex several joints well beyond the normal range, causing subluxations to different extents. JH is relatively common in certain categories of patients such as children where the prevalence ranges from 10 to 30 % [3], but also in adult women, who are more affected than men [3]. Some epidemiological surveys have reported the prevalence of generalized JHS in the general population as from 5 to 43 % depending on the assessment criteria, age, sex and ethnicity [3, 20–24]. Generalized JH is more common in infancy and decreases with age; however, musculoskeletal pain increases over time from childhood to adolescence and through adult life [25]. Estimating the frequency of JHS in later life is difficult because the diagnosis is complicated by physiological changes in connective tissue extensibility during aging. This markedly reduces articular hyper-extension, masking the syndrome. This explains why some studies report a JHS frequency similar to a rare disease (1/5000) [26] while recent findings suggest a significant increase in the prevalence that could affect from 0.75 to 2 % of the general population [27].

The clinical picture of JHS involves variable manifestations mainly but not exclusively in the musculoskeletal system, and evolving over the person’s lifetime. Trauma, pregnancy or any condition that obliges the patient to remain bedridden for any length of time can trigger the onset of the syndrome - as in our patient whose symptoms worsened after two consecutive infections and a neck trauma.

Even if arthralgias and myalgias are the most common pain presentation in young adults [25], frequently there are also cramps, enthesopathies, tendonitis, synovitis, bursitis and fasciitis [28]. Generalized muscle hyperalgesia, a true fibromyalgic syndrome or a chronic pain syndrome are common additional features (rev in [12]). Our patient had had joint instability since childhood (ability to do the splits, patellar instability, recurrent sprains to hands and ankles) but only in adolescence complained of progressive musculoskeletal disorders, in the form of recurrent tendonitis, joint instability, diffuse myalgias and pain that led to the diagnosis of fibromyalgia at first and subsequently to the suspicion of neurological or primary muscle disease. The pathogenesis of musculoskeletal pain can be related to joint instability which facilitates repeated microtrauma to articular and periarticular structures [28]. The laxity of tendino-ligamentous tissues produces a lack of muscle tone which causes defective proprioception and muscle deconditioning [29]. This is closely related to the impaired body image perception which leads to wrong postures, aggravating the damage to the musculoskeletal apparatus during everyday life and sport [28].

Even though this pathological circuit seems pivotal to the rise and establishment of muscular symptoms, a subsequent centralization of pain has been recently suggested in JHS patients [12]. Dysesthesias and peripheral paresthesias are recurrent complaints [30], such as our patient had developed during the last few months before our evaluation. Skin laxity is a frequent clinical sign [14, 31] and can be demonstrated by exceptional extensibility on the dorsum of the hands or on the elbows.

Fatigue and faintness are other common findings in the widespread clinical picture of JHS [32] that strongly limit physical activities, with a heavy impact on everyday life - as in our case. In fact, it has been recently reported that 84 % of JHS patients (mostly females) present weakness but the physiopathological basis remains elusive up to the present day [32]. Sleep disturbance [4, 32], poor postural control with consequent anti-gravitational muscle over-activation and in some cases autonomic alterations (palpitation, postural orthostatic hypotension and tachycardia) [6, 33, 34] have been suggested as possible concomitant causes of asthenia in affected people.

JHS is also frequently associated with visceral pain and gastrointestinal disturbance [8, 9]. For instance, our patient sometimes alternates several episodes of diarrhea in a day with some days of complete constipation, when she suffers abdominal discomfort. The pathogenesis of the visceral pain and the dysfunctional features is less understood, though weakness in the supporting structures, such as the abdominal wall and pelvic floor, are probably responsible for visceral ptosis, elongation and abnormal dilatation which may be the anatomical counterparts of a more complex functional gastrointestinal derangement [12].

Diagnosing JHS may pose a real challenge for the clinician who is not familiar with the numerous manifestations of this syndrome. The clinical manifestations go beyond the confines of the musculoskeletal system [10]. The main presenting complaints are nociceptive pain such as arthralgias, myalgias and back pain [25], often associated with a history of sprains and joint instability. Other features are abdominal pain [8, 9], headache [34], fatigue [32] but also dysmenorrhea, fibromyalgia and sleep disturbance [4, 32].

It is essential to read between the lines of complaints to distinguish other clinical situations and even psychiatric conditions such as hypochondria. Diseases that have generalized joint laxity as a clinical feature, like MFS or EDS, must be included in the differential diagnosis of JHS but each of these disorders has several features which distinguish it from JHS. An exception is the EDS-HT subtype which has almost the same clinical spectrum; in fact, these two conditions are sometimes considered as different degrees of the same syndrome [10].

The fact that this syndrome is not well known may result in unnecessary examinations and harmful treatments. Our patient was prescribed clonazepam for more than a year with no useful results. She also had standard physiotherapy which only worsened the condition. For these reasons JHS is not only a significant cause of discomfort for patients but also a great challenge for clinicians and a heavy burden on health systems. A useful approach to detect JHS promptly in patients with arthralgia, prone to recurrent articular injuries, could be based on correct administration of the Hakim and Grahame questionnaire (Table 2) as a first step, followed by the Brighton diagnostic criteria (Table 1).

The management of JHS can be very challenging and is currently hampered by the paucity of large cohort comparative data on treatments [3, 12, 35]. While judicious use of non-steroidal anti-inflammatory drugs can be useful to relieve joint and muscle pain, non-pharmacologic interventions are the core in the management of joint hypermobility and JHS [36]. A physiotherapist with special interest in joint hypermobility is recommended for patient education, strengthening exercises, core stability, teaching posture, joint protection and selected use of braces and orthoses [27]. A podiatric assessment is also suggested to improve biomechanical and support of the feet. Very useful recommendations for the management of musculoskeletal pain and fatigue in JHS were recently published by Castori et al. [12]. Nevertheless, it is cardinal importance for clinicians to refer these patients to specialized centers where a multidisciplinary approach and a patient education program can be applied to treat the various manifestation of the syndrome. Some useful information for readers can be found in HMSA (http://hypermobility.org) and EDSUK (www.ehlers-danlos.org) support groups.

Our patient was referred to a specialized hypermobility unit in the city where she was studying. The diagnosis was confirmed and the patient was placed in a coordinated program of physiotherapy, pain management and podiatry. Treatment focused on regaining muscle tone and improving proprioception without excessive joint stress. At more than 1 year treatment is still in progress and the patient reported a considerable positive impact on her quality of everyday life even after the few first sessions of physical reconditioning.

In conclusion, this case report highlights the clinical complexity and the multidisciplinary importance of JHS, an unexpectedly common disease that still tends to be under-recognized. Early diagnosis is essential to avoid long and often unnecessary diagnostic paths and to define not only the chronic pain but also the complex systemic symptoms that often characterize the syndrome. Management of JHS is equally intricate and requires a coordinated intervention that includes patient education, personalized physiotherapy and multidisciplinary medical collaboration.

### Consent

Written informed consent was obtained from the patient for publication of this case report and any accompanying images. A copy of the written consent is available for review by the Editor of this journal.

## Abbreviations

JH: joint hypermobility
MFS: Marfan syndrome
EDS: Ehlers-Danlos syndrome
JHS: joint hypermobility syndrome
EDS-HT: EDS hypermobility type
